# Assessing the Quality of Mobile Phone Apps for Weight Management: User-Centered Study With Employees From a Lebanese University

**DOI:** 10.2196/mhealth.9836

**Published:** 2019-01-23

**Authors:** Marco Bardus, Ahmed Ali, Farah Demachkieh, Ghassan Hamadeh

**Affiliations:** 1 Department of Health Promotion and Community Health Faculty of Health Sciences American University of Beirut Beirut Lebanon; 2 SANAD - The Home Hospice Organization of Lebanon Beirut Lebanon; 3 Department of Family Medicine Faculty of Medicine American University of Beirut Beirut Lebanon

**Keywords:** mobile apps, weight loss, physical activity, healthy diet, workplace, mHealth

## Abstract

**Background:**

Evaluating the quality of mobile health apps for weight loss and weight management is important to understand whether these can be used for obesity prevention and treatment. Recent reviews call for more research on multidimensional aspects of app quality, especially involving end users, as there are already many expert reviews on this domain. However, no quantitative study has investigated how laypersons see popular apps for weight management and perceive different dimensions of app quality.

**Objective:**

This study aimed to explore how laypersons evaluate the quality of 6 free weight management apps (*My Diet Coach*, *SparkPeople*, *Lark*, *MyFitnessPal*, *MyPlate*, and *My Diet Diary*), which achieved the highest quality ratings in a related and recent expert review.

**Methods:**

A user-centered study was conducted with 36 employees of a Lebanese university. Participants enrolled in the study on a rolling basis between October 2016 and March 2017. Participants were randomly assigned an app to use for 2 weeks. App quality was evaluated at the end of the trial period using the Mobile App Rating Scale user version (uMARS). uMARS assesses the dimensions of *engagement*, *functionality*, *aesthetics*, *information*, and *subjective quality* on 5-point scales. Internal consistency and interrater agreement were examined. The associations between uMARS scores and users’ demographic characteristics were also explored using nonparametric tests. Analyses were completed in November 2017.

**Results:**

Overall, the 6 apps were of moderately good quality (median uMARS score 3.6, interquartile range [IQR] 0.3). The highest total uMARS scores were achieved by *Lark* (mean 4.0 [SD 0.5]) and *MyPlate* (mean 3.8 [SD 0.4]), which also achieved the highest subjective quality scores (*Lark*: mean 3.3 [SD 1.4]; *MyPlate*: mean 3.3 [SD 0.8]). *Functionality* was the domain with the highest rating (median 3.9, IQR 0.3), followed by *aesthetics* (median 3.7, IQR 0.5), *information* (median 3.7, IQR 0.1), and *engagement* (median 3.3, IQR 0.2). *Subjective quality* was judged low (median 2.5, IQR 0.9). Overall, *subjective quality* was strongly and positively related (*P*<.001) with total uMARS score (ρ=.75), *engagement* (ρ=.68), *information*, and *aesthetics* (ρ=.60) but not *functionality* (ρ=.40; *P*=.02). Higher *engagement* scores were reported among healthy (*P*=.003) and obese individuals (*P*=.03), who also showed higher total uMARS (*P*=.04) and *subjective quality* (*P*=.05) scores.

**Conclusions:**

Although the apps were considered highly functional, they were relatively weak in engagement and subjective quality scores, indicating a low propensity of using the apps in the future. As engagement was the subdomain most strongly associated with subjective quality, app developers and researchers should focus on creating engaging apps, holding constant the functionality, aesthetics, and information quality. The tested apps (in particular *Lark* and *MyPlate*) were perceived as more engaging and of higher quality among healthy, obese individuals, making them a promising mode of delivery for self-directed interventions promoting weight control among the sampled population or in similar and comparable settings.

## Introduction

### Background

Mobile health (mHealth) apps offer cost-efficient and effective strategies to prevent noncommunicable diseases such as obesity or diabetes [1], as these technologies can reach millions of users. According to the 2017 mHealth App Economics report, there are more than 350,000 health apps available in online stores [2], a market worth US $25 billion in 2017 [3] and estimated to reach US $31 billion by 2020 [4]. mHealth apps are generally designed for chronically ill people (56%), fitness enthusiasts (33%), and physicians (32%) [4], with users downloading them with the aim to monitor their fitness and track foods as well as to manage chronic conditions [5]. A recent study specifically evaluating the market of weight management apps in 10 different countries [6] identified 28,905 unique apps that focus on physical activity (34%); diet (31%); and on tracking exercise, calorie intake, and body weight (23%) [6].

Although the mHealth app market is expected to expand in the next 3 years [7], recent market research reports show a decline in app usage [4]. Some qualitative studies show that users stop using apps because of hidden costs, increased data entry burden [8], and low perceived engagement [9]. Engagement with an app is generally associated with sustained app usage [1], but it has also been associated with positive changes in physical activity [10,11] and diet [12], fundamental behaviors to obtain an optimal weight management. Understanding which apps are perceived engaging and of good quality is important to develop effective public health strategies addressing these problems [3]. The more people use the apps they like, the more likely people will perform the desired behaviors.

Are mHealth apps effective? Several recent systematic reviews suggest that mobile phone apps are effective in promoting dietary self-regulation [13] and weight management [14-20]. Despite lacking evidence-based content [6,21], health apps can be used as stand-alone delivery modes in *self-directed* weight loss interventions [22,23] or as supplemental components of complex interventions. Some studies employing researcher- developed apps [24] or popular calorie counting apps (eg, *MyFitnessPal* [25,26]) in combination with face-to-face delivery modes showed generally larger effects compared with interventions using the apps as standalone [27-29].

How do these apps work? According to several app audits or reviews, mobile phone apps include features that can trigger cognitive processes underpinning effective behavior change strategies or techniques [30-35], combining principles derived from self-determination theory [22,23] and persuasive technology [36,37]. For example, apps may include messages or notifications that remind users about their weight goals and provide positive feedback or reinforcements for achieving those goals. In a recent review of 23 popular weight management apps [30], researchers found that most apps included several change techniques that are commonly employed in effective behavior change interventions. The most frequently identified change techniques were self-monitoring of behavior (20/23, 87%), self-monitoring and goal setting of outcomes (both 19/23, 83%), feedback on outcomes (17/23, 74%), feedback on behavior (16/23, 70%), and goal setting of behavior (13/23, 57%) [30]. Although research demonstrated the efficacy of these techniques in influencing behavior, available evaluations of app quality cannot demonstrate app efficacy. Assessing app quality has become an important stream of research, with several authors arguing for the need to improve the quality evaluation and the need to use standardized tools and systematic approaches [38]. However, expert app evaluations or reviews do not take into account the point of view of end users. Little is known about how end users perceive the apps and in what terms they judge their quality.

In a recent review on app quality assessment methods [39], the authors emphasized the need to use multidimensional tools to comprehensively determine the quality of mobile phone apps, which should also include end users’ viewpoints. This is because the views of researchers and end users tend to diverge. On one side, researchers focus on aspects related to theoretical and evidence-based content [38,39]. For example, in the aforementioned expert app review [30], the authors judged the 23 apps as highly functional but poor in information quality, lamenting the absence of references to evidence-based content. At the same time, their quality ratings were not significantly associated with the 5-star ratings derived from Google Play and iTunes stores, suggesting a potential gap between the wisdom of the crowds and of the experts [30]. App store ratings cannot be entirely trusted as these ratings can be piloted through reviews and ratings provided by humans or bots paid by the same developer companies [40]. On the other side, developers tend to focus on usability and aesthetic aspects, such as design, ease of use, and customizability, as some qualitative studies demonstrate that these aspects are particularly appreciated by end users [8,9,41].

One of the most comprehensive and multidimensional tools to evaluate app quality is the Mobile App Rating Scale (MARS). Developed by Stoyanov et al for expert reviews [42], the MARS has also been developed and validated for end users [43]. The MARS and the user version of the Mobile App Rating Scale, uMARS are multidimensional as they encompass the domains of *engagement*, *functionality*, *aesthetics*, and *information*, which are used to estimate an *objective* app quality dimension (calculated as an average score of the aforementioned domains), based on objective features and characteristics of an app. Each domain consists of a set of items, assessed on 5-point scales. The engagement domain includes 5 items: *entertainment*, *interest*, *customization*, *interactivity*, and *target group*. Functionality includes 4 items: *performance*, *ease of use*, *navigation*, and *gestural design*. Aesthetics includes 3 items: *layout,* *graphics*, and *visual appeal*. Information includes 4 core items: *quality*, *quantity*, *visual* information, as well as *credibility* of the source of information. The MARS scale includes 2 additional items: *accuracy of app description* and *goals* (ie, Does app have specific, measurable, and achievable goals specified in app store description or within the app itself?). The latter items, in fact, require additional information that a lay user might not easily find while using the app. Finally, both scales have also a *subjective quality* domain, which includes 4 items: Would you recommend this app to people who might benefit from it?; How many times do you think you would use this app in the next 12 months, if it was relevant to you?; Would you pay for this app?; and What is your overall star rating of the app? Due to the third item, it can be assumed that the higher the subjective quality score, the more likely the users would use the app in the future; however, the instrument does not include a measure of actual behavior (eg, “How many times have you used this app in the past day or week”). The MARS and uMARS tools are available from the respective MARS [42] and uMARS [43] development studies.

The MARS tool, generalized to primary prevention apps [44], has been used in several expert reviews of apps for a variety of behaviors such as drink driving [45], sustainable food consumption [46], medication adherence [47], mental health and mindfulness [48], quality of life [49], rheumatoid arthritis [50], weight loss related to smoking cessation [51], and weight management [30]. The user version, originally tested on 2 harm minimization and affect management apps [43], assessed the apps according to the same domains. The only differences between the 2 tools are wording of the questions and the number of items assessing the information domain. The uMARS use has been documented in research protocols of trials addressing type 2 diabetes [52], health-related quality of life [53], pneumococcal disease [54], and breastfeeding [55]. However, to the best of our knowledge, the uMARS tool has not been used to quantitatively evaluate commercially available weight management apps. In addition, little is known about what users believe are important app characteristics, that is, app quality dimensions and how these dimensions relate to the overall app quality. Furthermore, according to the leading author of the scale (Stoyanov S, personal communication, November 2017), the items belonging to each domain were logically grouped, but no MARS or uMARS studies to date appear to have evaluated the relationships among different app quality dimensions.

### Aims of the Study

In response to the call for more research on app quality evaluations from end users [39], the overarching goal of this study was to explore how laypersons evaluate the quality of a set of weight management apps, which experts considered of high quality in a recent review [30]. Specifically, this study aimed to (1) test the uMARS within a set of weight management apps; (2) understand which dimensions of app quality contribute the most to the overall app quality and how functionality, aesthetics, engagement, and information dimensions are related to subjective quality (as proxy of future app use); and (3) explore the associations between uMARS scales and users’ characteristics.

## Methods

### App Selection

A user experience study was used to examine the perceived quality and usability of selected apps and identify which apps achieve the best quality scores, which could be used in further studies with the same target population (employees of an academic institution). The units of analysis of this study were derived from a recent review of mobile phone apps for weight management [30]. In the cited review, only 6 out of the 23 apps reviewed (Table 1) scored above the median point of the MARS scale (3 out of 5), which is the median value of a 5-point scale. This value has been considered the minimum threshold of acceptability in the study by Mani et al [56].

### Participants and Procedures

Following recommendations from user experience and usability testing literature [57,58], we aimed to recruit 5 to 6 evaluators per app (30-36 participants). Participants were employees (faculty and staff) of the American University of Beirut, who were recruited through social media postings and email invitations (the research team obtained a list of randomly selected email addresses).

**Table 1:** List of apps used in the study, sorted by total Mobile App Rating Scale score, with app store information.

App name	Total MARS^a^ score^b^	Google Play rating^c^ (n)	iTunes rating^c^ (n)
*My Diet Coach*	4.6	4.6 (20,115)	4.6 (6040)
*SparkPeople*	4.4	4.4 (30,453)	4.6 (3677)
*Lark*	4.1	4.1 (2940)	4.1 (4294)
*MyFitnessPal*	3.9	4.6 (1,701,093)	4.7 (621,127)
*MyPlate*	3.5	4.6 (18,085)	4.6 (18,688)
*My Diet Diary*	3.4	4.1 (18,415)	4.2 (1280)

^a^MARS: Mobile App Rating Scale.

^b^Derived from the expert review by Bardus et al [30].

^c^Average 5-star rating and total number of ratings based on all versions of the app, as of November 15, 2017.

Interested employees submitted an informed consent and completed a Web-based eligibility survey. Inclusion criteria were participants aged 18 to 65 years, employees of the university, and owning either Android or iPhone devices. After enrollment and after signing an informed consent, which included all study schedules and requirements, participants completed a Web-based sociodemographic and behavioral baseline survey. Then, they were randomly assigned to use 1 of the apps for 2 weeks. A member of the research team helped each participant install the assigned app and verified that it was correctly installed and functioning. The same member of the research team encouraged participants to use the app at least daily for the duration of 2 weeks. At the end of this study period, they were invited to complete a final Web-based app evaluation survey. They received US $10 to complete each survey. The study was approved by the local institutional review board (reference number FHS.MB.01) and was conducted between October 2016 and March 2017; analyses were completed in November 2017.

### Measures

#### Background Characteristics

Background characteristics of the users included sociodemographic (age, gender, marital status, education, income, and number of working hours), health-related, and behavioral factors (perceived health status, height and weight, and physical activity assessed through the International Physical Activity Questionnaire-short form) [59]. App usage characteristics included operative system (Android or iOS) and previous experience with mHealth apps (for physical activity, diet, or weight tracking).

#### Quantitative Outcomes

App quality was evaluated employing the uMARS tool [43], which includes 20 items, as described in the introduction. The items are grouped into 4 *objective* subdomains: *engagement* (5 items), *functionality* (4), *aesthetics* (3), *information* (4), and 1 additional domain of *subjective quality* (4). *Subjective quality* scale includes 4 items that assess the intention to use the app in the future (ie, “Would you recommend this app to people who might benefit from it?” and “How many times do you think you would use this app in the next 12 months if it was relevant to you?”), propensity to pay for it (“Would you pay for this app?”), and an overall 5-star rating (“What is your overall star rating of the app?”), which reflects the way app stores rate the apps. All uMARS items are assessed through 5-point scales. Subscales are computed by averaging the respective domain items. A total uMARS score is calculated by averaging all subdomains, whereas *subjective quality* is calculated by averaging its related subitems. In the source study, the uMARS tool showed good internal consistency (Cronbach alpha=.90) and good test-retest reliability [43].

### Data Analyses

Survey data were summarized using descriptive statistics. Background characteristics were kept continuous (age), dichotomous (gender), or categorical (height and weight were used to compute body mass index, BMI). Following the International Physical Activity Questionnaire scoring protocol, physical activity was categorized as high, moderate, or low [59]. For uMARS items, answers categorized by users as “don’t know/not applicable” were coded as missing. Missing value analysis was performed to estimate the frequency and level of missingness and determine the best strategy to address the issue (eg, multiple imputation [MI] and listwise deletion). Internal consistency (Cronbach alpha) was interpreted as excellent (≥.90), good (.80-.89), acceptable (.70-.79), questionable (.60-.69), poor (.50-.59), and unacceptable (<.50) [44].

As each app was evaluated by different groups of users, traditional interrater reliability (IRR) indices (ie, intraclass correlation coefficients, ICCs), reported in MARS and uMARS development studies, were not applicable [60,61]. To ensure that ratings could be aggregated, we evaluated interrater agreement (IRA) following literature recommendations [62,63], using 3 families of indices: James et al’s *r*_*WG(J)*_ [64,65] (based on multiple null distributions) [66], Brown et al’s *a*_*WG(J)*_ [67], and the adjusted average deviation index *A*_*DMJ(adj)*_ [68]. IRA was established with pragmatic and theoretical cut-off points such as for the *r*_*WG(J)*_: no agreement (<.29), weak (.30-.49), moderate (.50-.69), strong (.70-.89), and very strong (>.90) [64,65]; *a*_*WG(J)*_: not acceptable (<.59), weak (.60-.69), moderate (.70-.79), and strong agreement (>.80) [67]; and *A*_*DMJ(adj)*_: agreement above .80 [68]. Strong agreement was considered when all indices were consistently indicating an acceptable level of agreement.

In addition to the arithmetic mean of each uMARS score, we calculated a response data–based weighted mean (WDMEAN) [69]. The WDMEAN allows to incorporate individual raters’ disagreements as it is calculated as the sum of each individual score multiplied by its weight, which is a function of the distance of the individual response from the unweighted group mean. This aggregation approach has been employed in organizational and management literature to summarize opinions from key informants who may not share the same knowledge about the object of study [70,71] and have some expected disagreement [69,70,72]. Unweighted and weighted mean scores (range: 1-5) were expressed as percent scores. The scale midpoint (3, converted in percent, assuming that 1=0%, 5=100%, and 3=50%) was considered the minimum level of acceptability, as reported in the study by Mani et al [56]. The WDMEAN, in presence of full agreement, would correspond to the arithmetic mean.

Considering the small sample size and the nature of the scores (which might be prone to non-normal distribution), associations among and with uMARS domain scores were examined by inspecting Spearman rho (ρ) coefficients. Total and uMARS subdomains were associated with subjective quality, as the associations among uMARS subdomains are not considered meaningful [43] or interpretable (Stoyanov S, personal communication, November 2017). Given the multiple tests, *P* values were corrected for type 1 error [73]. Mann-Whitney and Kruskal-Wallis (K-W) tests examined differences in continuous variables. Due to the exploratory nature of the study, no inferential statistics were attempted. All analyses were performed with IBM SPSS Statistics v.24 for Mac.

## Results

### Participant Recruitment

Invitations were sent to 600 randomly selected email addresses, and additional 145 employees were recruited through social media postings. Out of 745 potentially interested employees, 44 provided informed consent and 5 were ineligible. The remaining 39 employees successfully enrolled in the study. Moreover, 36 of them completed the app evaluations and were included in the analyses. Their characteristics are reported in Table 2. Employees were on average 36 years old (SD 10.8), mostly female (24/36, 67%), married (19/36, 53%), with a graduate-level university degree (16/36, 44%), earned less than US $2000 per month (17/36, 47%), and worked on average 48 hours per week (SD 11.9). The majority reported being in very good or excellent health status (16/36, 44%), normal weight (17/36, 47%), or overweight (16/36, 44%), and moderately active (28/36, 78%), spending on average 6.6 hours per day (SD 2.4) sitting. Most users owned an iOS device (21/36, 58%), and some had previously used apps for tracking physical activity (22/36, 60%), diet (8/36, 23%), or weight (4/36, 11%). A total of 6 participants had previously used 1 of the reviewed apps (*MyFitnessPal*). Group allocation was not associated with any background characteristic.

### App Quality Evaluation

Of the 36 users, 14 (39%) provided complete data covering 91% of values across the 20 uMARS items. The highest proportion of missingness was in the 3 *information* items (*credibility of source*: 39%; *visual information*: 25%; and *quantity of information*: 22%) and in 1 *engagement* item (*customization*: 19%). As missing was completely at random (Little’s missing completely at random test: *χ*^2^_264_=251.8; *P*=.69), MI was employed. We generated 10 complete datasets [74,75] and ran the analyses with both incomplete and complete datasets to ensure comparability of results. For clarity and accuracy, all uMARS scores presented here are based on pooled means and variance estimates obtained from the MI datasets.

Internal consistency and IRA estimates are reported in Multimedia Appendix 1. Overall, Cronbach alpha values varied across the uMARS subdomains, being acceptable for *engagement* (alpha=.75) and *aesthetics* (alpha=.71), questionable for *functionality* (alpha=.61), poor for *information* (alpha=.51), and good for *subjective quality* (alpha=.88). Within each app, alphas were good for *subjective quality* (median .82, range .74 [*My Diet Diary*] to .93 [*Lark*]), acceptable for *engagement* (median .71, range .46 [*My Diet Coach*] to .93 [*MyFitnessPal*]) and *aesthetics* (median .70, range .42 [*Lark*] to .86 [*SparkPeople*]), and unacceptable for *information* (median .23, range .15 [*SparkPeople*] to .46 [*MyPlate*]). Negative alpha values were found among *engagement* and *information* items (S*parkPeople* and *MyFitnessPal* groups, respectively), indicating negative correlations among those items. IRA indices suggested overall agreement among users in most subdomains and for most apps. Moderate to strong agreement was found in *functionality* and *aesthetics* (all apps), whereas low agreement was found in *engagement* (*MyFitnessPal* and *My Diet Diary*), *information* (*My Diet Diary*, *MyPlate*, and *SparkPeople*), and *subjective quality* (*Lark*, *MyFitnessPal*, and *My Diet Diary*).

**Table 2:** Characteristics of study participants according to app group and total sample (n=36).

Participants’ characteristics			*Lark* (n=7)	*MyFitnessPal* (n=6)	*My Diet Coach* (n=6)	*My Diet Diary* (n=5)	*MyPlate* (n=6)	*SparkPeople* (n=6)	Total sample (n=36)	*P* value
**Sociodemographics**										
	Age (years), mean (SE)		39.7 (5.3)	41.5 (3.8)	29.8 (2.6)	31.2 (4.5)	38.7 (4.4)	31.5 (4.1)	35.6 (1.8)	.14
	Gender (female), n (%)		2 (29)	3 (50)	5 (83)	4 (80)	5 (83)	5 (83)	24 (67)	.19
	**Marital status, n (%)**									.95
		Single	1 (14)	2 (33)	2 (33)	1 (20)	2 (33)	3 (50)	11 (31)	
		Engaged or in a relationship	1 (14)	1 (17)	1 (17)	2 (40)	1 (17)	0 (0)	6 (17)	
		Married	5 (71)	3 (50)	3 (50)	2 (40)	3 (50)	3 (50)	19 (53)	
	**Education, n (%)**								.06
		High school (secondary)	0 (0)	1 (17)	0 (0)	0 (0)	0 (0)	0 (0)	1 (3)	
		Bachelor	3 (43)	0 (0)	0 (0)	4 (81)	1 (17)	2 (33)	10 (28)	
		Master	3 (43)	2 (40)	3 (60)	0 (0)	5 (83)	3 (50)	16 (44)	
		PhD	1 (14)	3 (60)	2 (40)	1 (20)	0 (0)	1 (17)	8 (22)	
	**Income (n=33), n (%)**									.57
		<$US 2000	3 (43)	2 (33)	3 (50)	3 (60)	3 (50)	3 (50)	17 (47)	
		$US 2001 to $US 4000	0 (0)	2 (33)	1 (17)	1 (20)	2 (33)	3 (50)	9 (25)	
		>US $4000	2 (29)	2 (33)	2 (33)	1 (20)	0 (0)	0 (0)	7 (19)	
	Working hours per week (n=35), mean (SE)		46.7 (3.1)	43.3 (2.1)	45.8 (2.4)	38.0 (9.6)	45.0 (4.1)	35.0 (7.7)	42.8 (2.0)	.65
**Health and behavioral characteristics**										
	**Health status, n (%)**									.49
		Poor or fair	3 (43)	2 (33)	3 (50)	0 (0)	0 (0)	2 (33)	10 (28)	
		Good	1 (14)	2 (33)	0 (0)	2 (40)	4 (67)	1 (17)	10 (28)	
		Very good or excellent	3 (43)	2 (33)	3 (50)	3 (60)	2 (33)	3 (50)	16 (44)	
	**BMI^a^** **category, n (%)**									.32
		Normal weight	1 (14)	3 (50)	3 (50)	3 (60)	2 (33)	5 (83)	17 (47)	
		Overweight	4 (57)	2 (33)	3 (50)	2 (40)	4 (67)	1 (17)	16 (44)	
		Obese and morbidly obese	2 (29)	1 (17)	0 (0)	0 (0)	0 (0)	0 (0)	3 (8)	
	**Activity level, n (%)^b^**									.32
		High	3 (43)	0 (0)	2 (33)	1 (20)	2 (33)	0 (0)	8 (22)	
		Moderate	4 (57)	6 (100)	4 (67)	4 (80)	4 (67)	6 (100)	28 (78)	
	Sitting time (hours per day; n=35), mean (SE)		7.1 (0.8)	6.7 (0.3)	8.4 (1.4)	6.8 (0.1)	4.4 (1.2)	6.2 (0.6)	6.6 (0.4)	.14
**Mobile phone use and mobile health (mHealth) app use**										
	Operative system (iOS), n (%)		3 (43)	5 (83)	5 (83)	2 (40)	4 (67)	2 (33)	21 (58)	.29
	**Past experience with mHealth apps (n=35)^c^, n (%)**									
		Used apps to track physical activity	3 (43)	6 (100)	4 (67)	2 (40)	4 (67)	2 (33)	21 (60)	.18
		Used apps to track diet	3 (43)	0 (0)	3 (50)	0 (0)	2 (33)	0 (0)	8 (23)	.09
		Used apps to monitor weight	0 (0)	1 (17)	2 (33)	0 (0)	1 (17)	0 (0)	4 (11)	.34
		Never used mHealth apps	1 (14)	0 (0)	2 (33)	3 (60)	2 (33)	4 (67)	12 (34)	.12
	**Use of listed apps in the past 6 months (n=6)^c^, n (%)**									
		*MyFitnessPal*	2 (29)	1 (17)	1 (17)	0 (0)	2 (33)	0 (0)	6 (17)	.64

^a^BMI: body mass index.

^b^Categorization based on the International Physical Activity Questionnaire scoring protocol [59].

^c^Multiple choice questions. *P* values represent the significance level of chi-square test (categorical variable) or Kruskal-Wallis test (continuous variables).

The unweighted, WDMEANs, and percent scores are presented in Table 3. Unweighted and WDMEANs were practically the same, with the former being generally lower than the latter. *Information* was the domain with the largest difference between unweighted and WDMEAN (1.3%), followed by *engagement* and *functionality* (both 1%), *aesthetics* and total uMARS score (0.1%). *Subjective quality* scores were also similar, with the highest difference in *Lark* (−2.4%).

**Figure 1:**
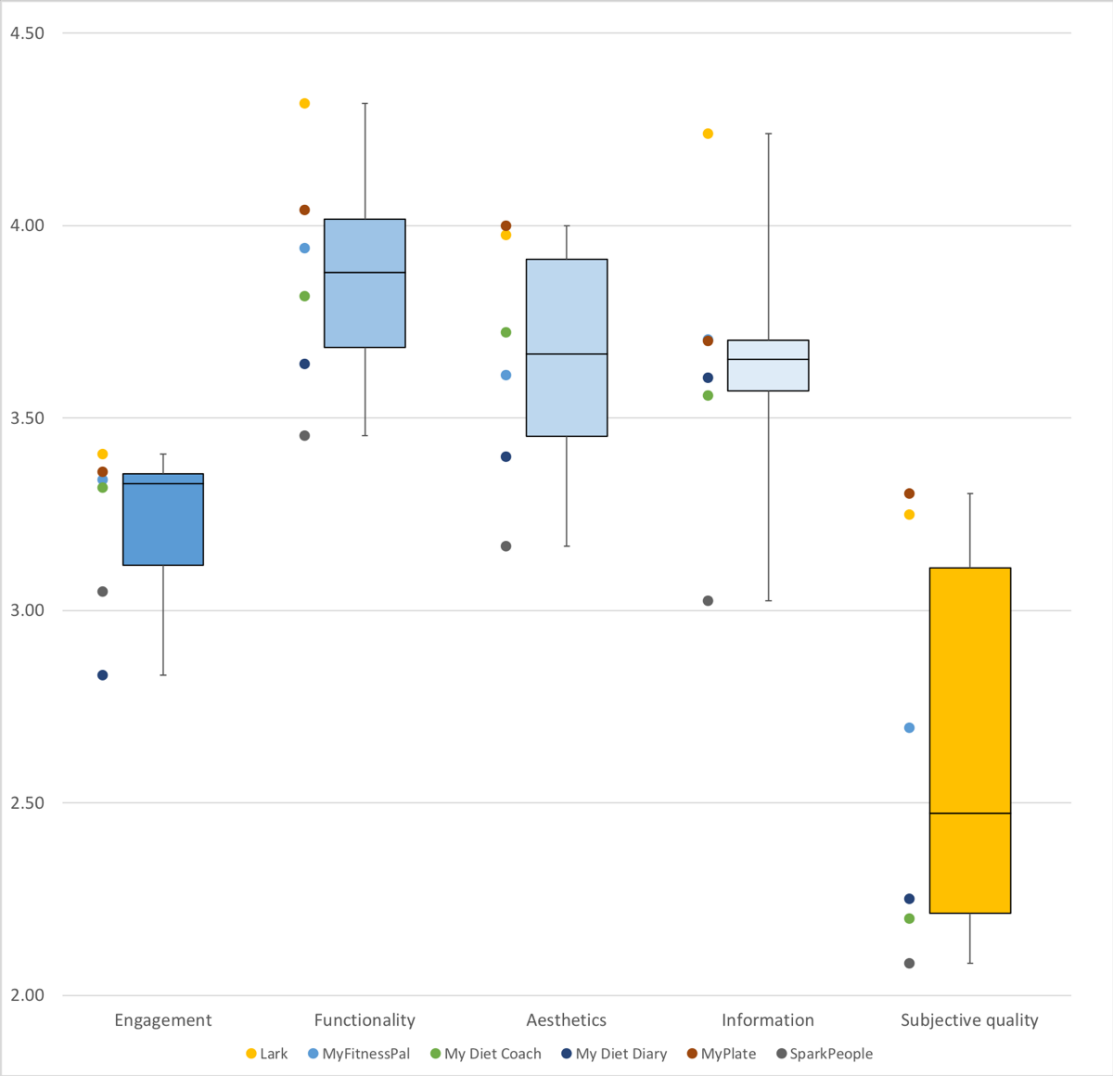
Boxplots of user version of the Mobile App Rating Scale subdomains and subjective quality with scatterplot representing each app.

Overall, all apps scored above the minimum threshold for acceptability (50%) in the total uMARS score and its main 4 subdomains. *Functionality* was the highest rated domain (median 3.9, interquartile range [IQR] 0.3), followed by *aesthetics* (median 3.7, IQR 0.5), *information* (median 3.7, IQR 0.1), and *engagement* (median 3.3, IQR 0.2). The *subjective quality* score was low (median 2.5, IQR 0.9). The scores are presented in the boxplot below (Figure 1). Only 2 apps (*MyPlate* and *Lark*) scored above the median thresholds in both uMARS and subjective quality scores.

After applying the Bonferroni correction for *P* values (*P*=.01), *subjective quality* was strongly and positively related (*P*<.001) with total uMARS score (ρ=.75), *engagement* (ρ=.68), *information*, and *aesthetics* (ρ=.60) and not significantly related with *functionality* (ρ=.40; *P*=.02).

### Associations With Users’ Characteristics

Correlations with users’ background characteristics are reported in Table 4. After applying the appropriate *P* value corrections for multiple correlation tests [73], good health status was associated with *engagement*, total uMARS, and *subjective quality*; being obese with total uMARS score; and use of *Lark* with *functionality* and *information*. Very good or excellent health status was negatively related to *engagement*; use of *SparkPeople* was negatively related to *information*. K-W tests revealed significant differences across health status groups in *engagement* (*χ*^2^_2_=11.9; *P*=.003), total uMARS (*χ*^2^_2_=9.4; *P*=.009), and *subjective quality* (*χ*^2^_2_=8.1; *P*=.02). Participants in good health status had higher median scores than those of the other 2 groups. Similarly, the 3 BMI categories (normal, overweight, and obese) scored significantly different in *engagement* (*χ*^2^_2_=6.8; *P*=.03), *functionality* (*χ*^2^_2_=6.1; *P*=.05), total uMARS score (*χ*^2^_2_=6.6; *P*=.04), and *subjective quality* (*χ*^2^_2_=6.11; *P*=.05). Obese individuals had higher median scores than those of the other 2 groups. Finally, K-W tests showed significasent differences among app groups in *information* (*χ*^2^_5_=14.4, *P*=.01) and total uMARS score (*χ*^2^_5_=12.4; *P*=.03). Users of *Lark* reported larger median *information* and total uMARS scores than the other apps. In *Lark*, *subjective quality* was positively associated with *engagement* (ρ=.87; *P*=.007) and total app quality (ρ=.90; *P*=.006). In *SparkPeople*, *subjective quality* was positively related to *information* (ρ=.97; *P*<.001).

**Table 3:** Comparison of user-based unweighted and weighted user version of the Mobile App Rating Scale scores.

App quality domains		Mean (SD)	Percent mean score	WDMEAN^a^	Percent WDMEAN score
**Engagement**					
	*Lark*	3.41 (0.84)	68.2	3.37	67.4
	*MyFitnessPal*	3.34 (1.22)	66.8	3.40	68.0
	*My Diet Coach*	3.32 (0.58)	66.4	3.44	68.8
	*My Diet Diary*	2.83 (0.83)	56.6	2.86	57.2
	*MyPlate*	3.36 (0.53)	67.2	3.39	67.8
	*SparkPeople*	3.05 (0.39)	61.0	3.17	63.4
**Functionality**					
	*Lark*	4.32 (0.53)	86.4	4.39	87.8
	*MyFitnessPal*	3.94 (0.53)	78.8	4.00	80.0
	*My Diet Coach*	3.82 (0.51)	76.4	3.80	76.0
	*My Diet Diary*	3.64 (0.61)	72.8	3.63	72.6
	*MyPlate*	4.04 (0.49)	80.8	4.17	83.4
	*SparkPeople*	3.45 (0.54)	69.0	3.49	69.8
**Aesthetics**					
	*Lark*	3.98 (0.74)	79.6	3.99	79.8
	*MyFitnessPal*	3.61 (0.49)	72.2	3.64	72.8
	*My Diet Coach*	3.72 (0.65)	74.4	3.85	77.0
	*My Diet Diary*	3.40 (0.55)	68.0	3.35	67.0
	*MyPlate*	4.00 (0.42)	80.0	4.00	80.0
	*SparkPeople*	3.17 (0.81)	62.0	3.10	62.0
**Information**					
	*Lark*	4.24 (0.60)	84.8	4.31	86.2
	*MyFitnessPal*	3.70 (0.73)	74.0	3.79	75.8
	*My Diet Coach*	3.56 (0.64)	71.2	3.57	71.4
	*My Diet Diary*	3.61 (0.53)	72.2	3.60	72.0
	*MyPlate*	3.70 (0.79)	74.0	3.76	75.2
	*SparkPeople*	3.03 (0.87)	60.6	3.10	62.0
**Total score**					
	*Lark*	3.98 (0.50)	79.2	3.96	79.2
	*MyFitnessPal*	3.65 (0.55)	74.0	3.70	74.0
	*My Diet Coach*	3.60 (0.43)	71.6	3.58	71.6
	*My Diet Diary*	3.37 (0.38)	65.8	3.29	65.8
	*MyPlate*	3.78 (0.40)	76.2	3.81	76.2
	*SparkPeople*	3.17 (0.45)	64.2	3.21	64.2
**Subjective quality**					
	*Lark*	3.25 (1.40)	65.0	3.37	67.4
	*MyFitnessPal*	2.70 (1.04)	54.0	2.73	54.6
	*My Diet Coach*	2.20 (0.76)	44.0	2.20	44.0
	*My Diet Diary*	2.25 (0.66)	45.0	2.24	44.8
	*MyPlate*	3.30 (0.84)	66.0	3.27	65.4
	*SparkPeople*	2.08 (0.68)	41.6	2.08	41.6

^a^WDMEAN: response data–based weighted mean [69].

**Table 4:** Correlations between user version of the Mobile App Rating Scales and users’ background characteristics.

Participants’ characteristics			User version of the Mobile App Rating Scales					
			Engagement	Functionality	Aesthetics	Information	Total score	Subjective quality
**Sociodemographics**								
	Age (years)		−0.11	0.34^a^	−0.03	0.17	0.09	0.29
	Gender: female		−0.14	−0.05	−0.17	−0.18	−0.15	−0.16
	**Marital status**							
		Single	0.24	−0.19	0.06	−0.16	0.04	−0.06
		Engaged	−0.05	−0.04	−0.16	0.17	0.02	−0.04
		Married	−0.18	0.21	0.06	0.02	−0.05	0.09
	**Education**							
		High school	0.28	0.10	0.10	0.08	0.19	0.12
		Bachelor	−0.11	−0.01	−0.06	0.03	−0.01	−0.06
		Master	−0.05	−0.16	0.06	−0.09	−0.09	−0.19
		PhD	0.06	0.16	−0.06	0.04	0.04	0.24
	**Income**							
		<US $2000	0.14	0.09	−0.03	0.06	0.13	−0.11
		<US $3000	0.00	−0.23	−0.03	−0.07	−0.07	0.06
		<US $4000	−0.30	−0.12	−0.08	−0.06	−0.18	−0.20
		>US $4000	−0.02	0.19	−0.05	0.18	0.05	0.31
	Working hours per week		−0.10	0.17	0.15	0.12	0.10	0.08
**Health and behavioral characteristics**								
	**Health status**							
		Poor or fair	0.01	0.02	−0.03	−0.07	−0.05	−0.22
		Good	0.54^b^	0.23	0.36^a^	0.34^a^	0.50^b^	0.48^b^
		Very good or excellent	−0.49^b^	−0.23	−0.30	−0.25	−0.40^a^	−0.24
	**Body mass index**							
		Normal weight	0.11	−0.29	−0.11	−0.28	−0.17	0.01
		Overweight	−0.32	0.08	−0.11	0.13	−0.07	−0.23
		Obese	0.38^a^	0.37^a^	0.38^a^	0.27	0.43^b^	0.40^a^
	Activity level: high		0.17	−0.02	0.07	0.11	0.09	−0.05
	Sitting time (hours per day)		0.12	−0.11	−0.06	−0.06	−0.03	0.04
**Mobile phone use and mobile health (mHealth) app use**								
	Mobile operative system: iOS		0.17	−0.01	0.25	0.18	0.18	0.22
	**Past experience with mHealth apps**							
		Used apps to track physical activity	0.08	−0.13	−0.06	0.16	0.01	0.08
		Used apps to track diet	−0.16	0.26	0.05	0.22	0.10	−0.13
		Used apps to monitor weight	0.13	0.32	0.05	0.20	0.20	0.01
		Never used mHealth apps	−0.03	−0.07	−0.03	−0.29	−0.14	−0.05
	**App used in the study**							
		Used *Lark*	0.08	0.43^b^	0.35^a^	0.47^b^	0.42^a^	0.24
		Used *MyFitnessPal*	0.08	0.05	−0.09	0.07	0.04	0.05
		Used *My Diet Coach*	0.06	−0.07	0.03	−0.16	−0.09	−0.21
		Used *My Diet Diary*	0.17	0.02	−0.12	−0.03	0.04	−0.01
		Used *MyPlate*	0.10	0.11	0.24	0.19	0.22	0.30
		Used *SparkPeople*	−0.12	−0.33^a^	−0.30	−0.47^b^	−0.37^a^	−0.24

^a^*P*<.05.

^b^*P*<.001. With Bonferroni correction, the significance value becomes *P*<.0003.

## Discussion

### Principal Findings

This is the first study that explored how laypersons evaluated the quality of free and popular mobile phone apps for weight management, using the uMARS tool [43]. The tool showed acceptable internal consistency levels in most subdomains, except for *information* (alpha=.51). Heterogeneity in alpha values was found within each app group. In 2 cases (*SparkPeople* for *engagement* and *MyFitnessPal* for *information*), alphas assumed negative values, which indicate small, negative correlations among the items in those subscales and lack of consistency. The internal consistencies we found are below those reported in the uMARS source study [43] and below the levels commonly recommended by the literature, suggesting large measurement errors [76]. Low alphas might be because of the number of items and sample size [77]. In addition, users might have had different interpretations of the items as some IRA indices pointed to low or no agreement within *engagement* items (*MyFitnessPal* and *My Diet Diary*), *information* (*My Diet Diary*, *MyPlate*, and *SparkPeople*), and *subjective quality* (*Lark*, *MyFitnessPal*, and *My Diet Diary*). Although IRA does not imply reliability [62,63], low agreement suggests a large degree of subjectivity in evaluating the apps, which can be expected, as the users are supposed to be free to have their own opinions about the apps, based on their own characteristics and needs.

Furthermore, large item nonresponse rates were registered in the *information* domain (22%-39%). Some users might have misunderstood these items or might not have known how to answer, thus leaving them blank. The missing information might explain the poor consistency and low agreement estimates in this specific domain. Unfortunately, the uMARS source study does not provide solutions in case of poor internal consistency or low agreement [43], and other studies employing uMARS did not report such issues [54,55]. To account for these limitations, we calculated the WDMEAN [69], an approach that allowed to retain all items. Eventually, the unweighted and weighted means were very similar, suggesting that applying the uMARS scoring protocol can still yield robust results. Nevertheless, the uMARS tool should be generalized to weight management apps, with larger user populations. We also recommend exploring users’ perceptions about the items including qualitative methodologies such as the *think aloud method* [78].

In this study, we employed the WDMEAN approach to estimate the responses from our key informants who were asked to apply the uMARS tool without previous training. To the best of our knowledge, no uMARS and MARS studies have used this approach, employing users who have undergone some level of training. This is the first study that utilizes the tool for users. By employing the WDMEAN method, it is possible to estimate app quality while accounting for the respondents’ potential disagreements, hence providing a *truer* average score, which accounts for the response of each individual [69]. On the contrary, the arithmetic mean can be influenced by extreme values (either very low or very high scores), and at the same time, it might reduce the intrinsic variability among raters’ ratings. The WDMEAN approach can be applied to many other studies, with small samples, in which researchers are interested in estimating scores while accounting for the agreement or disagreement among raters.

The second objective was to understand which app quality dimensions (ie, engagement, functionality, aesthetics, and information) contributed the most to the overall app quality score. All apps scored high in *functionality*, followed by *aesthetics*, *information*, and *engagement*. This is consistent with some qualitative research suggesting that users appreciate functional and aesthetic characteristics [8,9,41]. This is also consistent with the findings reported in the expert review, upon which this study is based, as the apps were deemed highly functional and with limited information quality [30]. However, *engagement*, *aesthetics*, and *information* appeared to be strongly related with *subjective quality*, which includes questions that indicate the propensity of using the apps in the future (“Would you recommend...,” “Would you pay,” “How many times would you use it...?,” and “What is the overall star rating?”). This might indicate that users might not engage with these apps regardless of their good functional features. This is consistent with findings from qualitative studies, which show that users might stop using an app not because of technical features but rather because of low engagement or hidden costs [8,9]. Another important consideration was that in our study, *subjective quality* was only weakly correlated with *functionality* (ρ=.40; *P*=.02). Conversely, *engagement* had the strongest correlation with *subjective quality* (ρ=.68; *P*<.001). This might indicate that app engagement can play an important role in achieving sustained app usage [10,12]; however, future studies should be conducted to establish whether a causal link between engagement and future app use exists.

The third objective was to explore the associations between uMARS scales and users’ characteristics. In this sample, we found that obese users and those in good health status provided higher app quality ratings in *engagement*, total uMARS, and *subjective quality*. In other words, healthy, obese individuals perceived these apps particularly engaging and of high quality. As engagement is related to app usage [1], these individuals might be more likely to use the apps in the future. These findings are particularly suggestive, as these popular weight management apps (in particular, *Lark*, *MyFitnessPal*, and *MyPlate*) may be used in interventions addressing obesity prevention (healthy volunteers) and treatment (obese) [9]. Future research could test whether these apps, which had demonstrated having high behavior change potential [30], can effectively influence behavior and promote weight loss among overweight or obese individuals. This study informed the development of a self-directed weight control intervention, which targets the same population (clinical trial registry: NCT03321331).

### Limitations

The results of this study need to be interpreted bearing in mind its limitations. A major limitation is the design (noncrossover). For feasibility reasons (budget and time constraints), we could not ask all users to evaluate each app, hence allowing us to calculate IRR using ICC indices. To overcome this limitation, we employed methodological solutions that have never been employed in similar studies (ie, IRA estimates [60,62] and WDMEAN [69]). These solutions allowed us to ensure the robustness of the responses obtained from the employees recruited in this study. This solution is pragmatic and allows to be applied in real-life scenarios, whereby research study participants might not be willing or able to dedicate more time to the study. Moreover, users evaluated the free version of the apps used for 2 weeks. Ratings might have differed if they had used the *pro* versions with additional functionalities. App evaluation might also be influenced by actual app use and by the amount of time spent on each app. As the authors of the expert review noted [30], some apps prompt different feedback and unlock features only after repeated use. We instructed participants to use the apps at least daily for 2 weeks, but we did not assess actual app use. Another limitation is the sampling of this study as we had access to a convenience sample of employees from an academic institution in Lebanon, who voluntarily agreed to participate. Although we found correlations with health status and BMI categories, this study might not be generalizable to the entire population and to other cultural contexts and settings, as we recruited mostly female, educated, and healthy individuals. The small sample size is also another limitation; however, the size was based on pragmatic considerations and aligned with recommendations from the heuristic evaluation literature [57,58]. Larger samples should investigate whether these findings hold truth in different segments of the population. It will be practical to focus studies on specific segments of the population to increase the accuracy of the findings. Nevertheless, we believe the results are generalizable to similar academic institutions in Lebanon or in the Middle East region or who have similar employee populations, although the tested apps are available internationally. Another limitation is the use of self-reported data and self-administered Web-based surveys that are prone to missing data. We used Web-based tools because we wanted to avoid interviewer bias and we did not want to interfere with the users’ evaluations of the apps. We wanted the users to test the apps *in the wild* for 2 weeks, without specialized training, which is usually a prerequisite of expert reviews. We could have used interviewers to reduce data entry mistakes or inconsistencies, but we opted for self-administered Web-based forms to avoid interviewer bias. A related limitation is the presence of large amounts of missing data in some of the subdomains of the uMARS scale (eg *information* domain), which forced us to apply caution when interpreting the results. Although we employed modern techniques to deal with missing data, we cannot make strong assumptions on the reasons for the missing responses backed on data, as the instrument (Web-based survey) did not capture comments related to the uMARS scale. We recommend that future studies investigate how users respond to the survey and how they apply the answers. We have already suggested that qualitative techniques such as the *think aloud method* [78] could be applied to understand the thought processes that people use when answering questionnaires. These techniques would allow to identify potential pitfalls in the scale, hence improving its validity across cultures and sample populations.

### Conclusions

Across the 6 popular and free weight management apps analyzed in this study, *functionality* is the quality dimension that laypersons valued the most. However, *engagement* was strongly associated with *subjective quality*, a dimension that includes future app use. The higher the subjective quality and engagement, the more likely users might use the app. App developers and public health professionals should ensure that an app is both functional and engaging so that users will be more likely to use it. Future longitudinal studies are needed to ascertain this connection.

The tested apps (in particular *Lark* and *MyPlate*) were perceived as more engaging and of higher quality among healthy, obese individuals, making them promising modes of delivery for obesity prevention and treatment interventions.

From a methodological standpoint, the uMARS tool is a practical and feasible tool that can be used to assess app quality by laypersons without specialized training. However, further research is needed to establish its validity in the domain of weight management.

## Appendix

Multimedia Appendix 1 Internal consistency and interrater agreement (IRA) indices for the user version of the Mobile App Rating Scale scores.

## Abbreviations

BMI: body mass index
ICC: intraclass correlation coefficient
IQR: interquartile range
IRA: interrater agreement
IRR: interrater reliability
K-W: Kruskal-Wallis
MARS: Mobile App Rating Scale
mHealth: mobile health
MI: multiple imputation
uMARS: user version of the Mobile App Rating Scale
WDMEAN: response data–based weighted mean

## References

[1] Kao C, Liebovitz DM (2017). Consumer mobile health apps: current state, barriers, and future directions. PM R.

[2] Research 2 Guidance (2017). Mhealth app developer economics 2017 Internet.

[3] The Lancet (2017). Does mobile health matter?. Lancet.

[4] Research 2 Guidance (2016). Mhealth app developer economics 2016 Internet.

[5] Krebs P, Duncan DT (2015). Health app use among US mobile phone owners: a national survey. JMIR Mhealth Uhealth.

[6] Nikolaou CK, Lean ME (2017). Mobile applications for obesity and weight management: current market characteristics. Int J Obes (Lond).

[7] Research 2 Guidance (2015). mHealth App Market Sizing 2015-2020 Internet.

[8] Alnasser AA, Alkhalifa AS, Sathiaseelan A, Marais D (2015). What overweight women want from a weight loss app: a qualitative study on arabic women. JMIR Mhealth Uhealth.

[9] Tang J, Abraham C, Stamp E, Greaves C (2015). How can weight-loss app designers' best engage and support users? A qualitative investigation. Br J Health Psychol.

[10] Hoj TH, Covey EL, Jones AC, Haines AC, Hall PC, Crookston BT, West JH (2017). How do apps work? An analysis of physical activity app users' perceptions of behavior change mechanisms. JMIR Mhealth Uhealth.

[11] Turner-McGrievy GM, Beets MW, Moore JB, Kaczynski AT, Barr-Anderson DJ, Tate DF (2013). Comparison of traditional versus mobile app self-monitoring of physical activity and dietary intake among overweight adults participating in an mHealth weight loss program. J Am Med Inform Assoc.

[12] West J, Belvedere L, Andreasen R, Frandsen C, Hall P, Crookston B (2017). Controlling your “app”etite: how diet and nutrition-related mobile apps lead to behavior change. JMIR Mhealth Uhealth.

[13] Semper HM, Povey R, Clark-Carter D (2016). A systematic review of the effectiveness of smartphone applications that encourage dietary self-regulatory strategies for weight loss in overweight and obese adults. Obes Rev.

[14] Aguilar-Martínez A, Solé-Sedeño JM, Mancebo-Moreno G, Medina FX, Carreras-Collado R, Saigí-Rubió F (2014). Use of mobile phones as a tool for weight loss: a systematic review. J Telemed Telecare.

[15] Bardus M, Smith JR, Samaha L, Abraham C (2015). Mobile phone and web 2.0 technologies for weight management: a systematic scoping review. J Med Internet Res.

[16] Quelly SB, Norris AE, DiPietro JL (2016). Impact of mobile apps to combat obesity in children and adolescents: a systematic literature review. J Spec Pediatr Nurs.

[17] Riaz S, Sykes C (2015). Are smartphone health applications effective in modifying obesity and smoking behaviours? A systematic review. Health Technol.

[18] Flores Mateo G, Granado-Font E, Ferré-Grau C, Montaña-Carreras X (2015). Mobile phone apps to promote weight loss and increase physical activity: a systematic review and meta-analysis. J Med Internet Res.

[19] Bardus M, Smith JR, Samaha L, Abraham C (2016). Mobile and Web 2.0 interventions for weight management: an overview of review evidence and its methodological quality. Eur J Public Health.

[20] Schippers M, Adam PC, Smolenski DJ, Wong HT, de Wit JB (2017). A meta-analysis of overall effects of weight loss interventions delivered via mobile phones and effect size differences according to delivery mode, personal contact, and intervention intensity and duration. Obes Rev.

[21] Pagoto S, Schneider K, Jojic M, DeBiasse M, Mann D (2013). Evidence-based strategies in weight-loss mobile apps. Am J Prev Med.

[22] Tang JC, Abraham C, Greaves CJ, Nikolaou V (2016). Self-directed interventions to promote weight loss: a systematic review and meta-analysis. Health Psychol Rev.

[23] Tang J, Abraham C, Greaves C, Yates T (2014). Self-directed interventions to promote weight loss: a systematic review of reviews. J Med Internet Res.

[24] Fukuoka Y, Gay CL, Joiner KL, Vittinghoff E (2015). A novel diabetes prevention intervention using a mobile app: a randomized controlled trial with overweight adults at risk. Am J Prev Med.

[25] Hartman SJ, Nelson SH, Cadmus-Bertram LA, Patterson RE, Parker BA, Pierce JP (2016). Technology- and phone-based weight loss intervention: pilot RCT in women at elevated breast cancer risk. Am J Prev Med.

[26] Ipjian ML, Johnston CS (2017). Smartphone technology facilitates dietary change in healthy adults. Nutrition.

[27] Laing BY, Mangione CM, Tseng C, Leng M, Vaisberg E, Mahida M, Bholat M, Glazier E, Morisky DE, Bell DS (2014). Effectiveness of a smartphone application for weight loss compared with usual care in overweight primary care patients: a randomized, controlled trial. Ann Intern Med.

[28] Jospe MR, Roy M, Brown RC, Williams SM, Osborne HR, Meredith-Jones KA, McArthur JR, Fleming EA, Taylor RW (2017). The effect of different types of monitoring strategies on weight loss: a randomized controlled trial. Obesity (Silver Spring).

[29] Nollen NL, Mayo MS, Carlson SE, Rapoff MA, Goggin KJ, Ellerbeck EF (2014). Mobile technology for obesity prevention: a randomized pilot study in racial- and ethnic-minority girls. Am J Prev Med.

[30] Bardus M, van Beurden SB, Smith JR, Abraham C (2016). A review and content analysis of engagement, functionality, aesthetics, information quality, and change techniques in the most popular commercial apps for weight management. Int J Behav Nutr Phys Act.

[31] Direito A, Dale LP, Shields E, Dobson R, Whittaker R, Maddison R (2014). Do physical activity and dietary smartphone applications incorporate evidence-based behaviour change techniques?. BMC Public Health.

[32] Conroy DE, Yang C, Maher JP (2014). Behavior change techniques in top-ranked mobile apps for physical activity. Am J Prev Med.

[33] Cowan LT, Van Wagenen SA, Brown BA, Hedin RJ, Seino-Stephan Y, Hall PC, West JH (2013). Apps of steel: are exercise apps providing consumers with realistic expectations?: a content analysis of exercise apps for presence of behavior change theory. Health Educ Behav.

[34] Lyons EJ, Lewis ZH, Mayrsohn BG, Rowland JL (2014). Behavior change techniques implemented in electronic lifestyle activity monitors: a systematic content analysis. J Med Internet Res.

[35] Azar KM, Lesser LI, Laing BY, Stephens J, Aurora MS, Burke LE, Palaniappan LP (2013). Mobile applications for weight management: theory-based content analysis. Am J Prev Med.

[36] Fogg B, Fogg B, Eckles D (2007). The Future of Persuasion is Mobile. Mobile persuasion: 20 perspectives on the future of behavior change.

[37] Matthews J, Win KT, Oinas-Kukkonen H, Freeman M (2016). Persuasive technology in mobile applications promoting physical activity: a systematic review. J Med Syst.

[38] BinDhim NF, Hawkey A, Trevena L (2015). A systematic review of quality assessment methods for smartphone health apps. Telemed J E Health.

[39] Grundy QH, Wang Z, Bero LA (2016). Challenges in assessing mobile health app quality: a systematic review of prevalent and innovative methods. Am J Prev Med.

[40] Zhu H, Xiong H, Ge Y, Chen E (2015). Discovery of ranking fraud for mobile apps. IEEE Trans Knowl Data Eng.

[41] Solbrig L, Jones R, Kavanagh D, May J, Parkin T, Andrade J (2017). People trying to lose weight dislike calorie counting apps and want motivational support to help them achieve their goals. Internet Interv.

[42] Stoyanov SR, Hides L, Kavanagh DJ, Zelenko O, Tjondronegoro D, Mani M (2015). Mobile app rating scale: a new tool for assessing the quality of health mobile apps. JMIR Mhealth Uhealth.

[43] Stoyanov SR, Hides L, Kavanagh DJ, Wilson H (2016). Development and validation of the user version of the mobile application rating scale (uMARS). JMIR Mhealth Uhealth.

[44] Domnich A, Arata L, Amicizia D, Signori A, Patrick B, Stoyanov S, Hides L, Gasparini R, Panatto D (2016). Development and validation of the Italian version of the Mobile Application Rating Scale and its generalisability to apps targeting primary prevention. BMC Med Inform Decis Mak.

[45] Wilson H, Stoyanov SR, Gandabhai S, Baldwin A (2016). The quality and accuracy of mobile apps to prevent driving after drinking alcohol. JMIR Mhealth Uhealth.

[46] Sullivan RK, Marsh S, Halvarsson J, Holdsworth M, Waterlander W, Poelman MP, Salmond JA, Christian H, Koh LS, Cade JE, Spence JC, Woodward A, Maddison R (2016). Smartphone apps for measuring human health and climate Change co-benefits: a comparison and quality rating of available apps. JMIR Mhealth Uhealth.

[47] Santo K, Richtering SS, Chalmers J, Thiagalingam A, Chow CK, Redfern J (2016). Mobile phone apps to improve medication adherence: a systematic stepwise process to identify high-quality apps. JMIR Mhealth Uhealth.

[48] Rickard N, Arjmand H, Bakker D, Seabrook E (2016). Development of a mobile phone app to support self-monitoring of emotional well-being: a mental health digital innovation. JMIR Ment Health.

[49] Zini F, Reinstadler M, Ricci F, Giokas K, Bokor L, Hopfgartner F (2017). Increasing Quality of Life Awareness with Life-Logging. eHealth 360°.

[50] Grainger R, Townsley H, White B, Langlotz T, Taylor WJ (2017). Apps for people with Rheumatoid arthritis to monitor their disease activity: a review of apps for best practice and quality. JMIR Mhealth Uhealth.

[51] Patel R, Sulzberger L, Li G, Mair J, Morley H, Shing MN, O'Leary C, Prakash A, Robilliard N, Rutherford M, Sharpe C, Shie C, Sritharan L, Turnbull J, Whyte I, Yu H, Cleghorn C, Leung W, Wilson N (2015). Smartphone apps for weight loss and smoking cessation: quality ranking of 120 apps. N Z Med J.

[52] Goyal S, Lewis G, Yu C, Rotondi M, Seto E, Cafazzo JA (2016). Evaluation of a behavioral mobile phone app intervention for the self-management of type 2 diabetes: randomized controlled trial protocol. JMIR Res Protoc.

[53] Mareva S, Thomson D, Marenco P, Estal Muñoz V, Ott C, Schmidt B, Wingen T, Kassianos A (2016). Study protocol on ecological momentary assessment of health-related quality of life using a smartphone application. Front Psychol.

[54] Panatto D, Domnich A, Gasparini R, Bonanni P, Icardi G, Amicizia D, Arata L, Carozzo S, Signori A, Bechini A, Boccalini S (2016). An eHealth project on invasive Pneumococcal disease: comprehensive evaluation of a promotional campaign. J Med Internet Res.

[55] White BK, Martin A, White JA, Burns SK, Maycock BR, Giglia RC, Scott JA (2016). Theory-based design and development of a socially connected, gamified mobile app for men about breastfeeding (milk man). JMIR Mhealth Uhealth.

[56] Mani M, Kavanagh DJ, Hides L, Stoyanov SR (2015). Review and evaluation of mindfulness-based iPhone apps. JMIR Mhealth Uhealth.

[57] Nielsen J (1994). Heuristic evaluation. Usability Inspection Methods.

[58] Roher C (2014). Nielsen Norman Group.

[59] (2011). IPAQ Research Committee.

[60] Hallgren KA (2012). Computing inter-rater reliability for observational data: an overview and tutorial. Tutor Quant Methods Psychol.

[61] Gwet K (2012). Handbook of Inter-Rater Reliability: The Definitive Guide to Measuring the Extent of Agreement Among Multiple Raters, 3rd Edition. 3rd edition.

[62] O'Neill TA (2017). An overview of interrater agreement on likert scales for researchers and practitioners. Front Psychol.

[63] LeBreton JM, Senter JL (2007). Answers to 20 questions about interrater reliability and interrater agreement. Organ Res Methods.

[64] James LR, Demaree RG, Wolf G (1984). Estimating within-group interrater reliability with and without response bias. J Appl Psychol.

[65] James L, Demaree R, Wolf G (1993). rwg: an assessment of within-group interrater agreement. J Appl Psychol.

[66] Biemann T, Cole MS, Voelpel S (2012). Within-group agreement: on the use (and misuse) of rWG and rWG(J) in leadership research and some best practice guidelines. Leadersh Q.

[67] Brown RD, Hauenstein NM (2016). Interrater agreement reconsidered: an Alternative to the rwg indices. Organ Res Methods.

[68] Lohse-Bossenz H, Kunina-Habenicht O, Kunter M (2013). Estimating within-group agreement in small groups: a proposed adjustment for the average deviation index. Eur J Work Organ Psychol.

[69] Van Bruggen GH, Lilien G, Kacker M (2002). Informants in organizational marketing research: why use multiple informants and how to aggregate responses. J Mark Res.

[70] Wagner SM, Rau C, Lindemann E (2010). Multiple informant methodology: a critical review and recommendations. Sociol Methods Res.

[71] Leuffen D, Shikano S, Walter S (2012). Measurement and data aggregation in small-n social scientific research. Eur Polit Sci.

[72] Woehr DJ, Loignon AC, Schmidt PB, Loughry ML, Ohland MW (2015). Justifying aggregation with consensus-based constructs. Organ Res Methods.

[73] Curtin F, Schulz P (1998). Multiple correlations and Bonferroni's correction. Biol Psychiatry.

[74] Barnes SA, Lindborg SR, Seaman JW (2006). Multiple imputation techniques in small sample clinical trials. Stat Med.

[75] Graham JW, Olchowski AE, Gilreath TD (2007). How many imputations are really needed? Some practical clarifications of multiple imputation theory. Prev Sci.

[76] Tavakol M, Dennick R (2011). Making sense of Cronbach's alpha. Int J Med Educ.

[77] Sijtsma K (2009). On the use, the misuse, and the very limited usefulness of Cronbach's alpha. Psychometrika.

[78] Perski O, Blandford A, Ubhi H, West R, Michie S (2017). Smokers' and drinkers' choice of smartphone applications and expectations of engagement: a think aloud and interview study. BMC Med Inform Decis Mak.

