# Supplementary material for: Assessing the Quality of Mobile Phone Apps for Weight Management: User-Centered Study With Employees From a Lebanese University
Source: JMIR Mhealth Uhealth. 2019 Jan 23;7(1):e9836. doi: 10.2196/mhealth.9836 (PMC6364203; doi:10.2196/mhealth.9836)
Supplement: Multimedia Appendix 1 [file mhealth_v7i1e9836_app1.pdf]

Multimedia Appendix 2. Internal consistency and Inter-rater agreement (IRA) indices for the uMARS scores

			Inter-rater agreement		
		Cronbach's alpha	$r_{WG(J)}$	$a_{WG(J)}$	$A_{DM[J](adj)}$
Engagement	Overall	0.75			
	Lark	0.88	0.75	0.52	0.82
	MyFitnessPal	0.93	0.54	0.19	1.14
	MyDietCoach	0.46	0.79	0.62	0.65
	MyDietDiary	0.71	0.66	0.38	1.01
	MyPlate	0.63	0.86	0.74	0.56
	SparkPeople	-0.04	0.83	0.70	0.56
Functionality	Overall	0.61			
	Lark	0.49	0.88	0.60	0.52
	MyFitnessPal	0.67	0.86	0.66	0.48
	MyDietCoach	0.43	0.84	0.66	0.62
	MyDietDiary	0.85	0.87	0.76	0.59
	MyPlate	0.47	0.85	0.63	0.52
	SparkPeople	0.45	0.82	0.68	0.67
Aesthetics	Overall	0.71			
	Lark	0.42	0.75	0.44	0.80
	MyFitnessPal	0.74	0.91	0.83	0.48
	MyDietCoach	0.74	0.84	0.70	0.61
	MyDietDiary	0.67	0.88	0.79	0.45
	MyPlate	0.44	0.91	0.79	0.41
	SparkPeople	0.86	0.79	0.65	0.69
Information	Overall	0.51			
	Lark	0.23	0.74	0.38	0.77
	MyFitnessPal	-0.79	0.78	0.59	0.67
	MyDietCoach	0.31	0.72	0.48	0.86
	MyDietDiary	0.23	0.82	0.68	0.64
	MyPlate	0.46	0.67	0.39	0.82
	SparkPeople	0.15	0.67	0.41	0.94
Subjective quality	Overall	0.88			
	Lark	0.93	0.41	-0.07	0.77
	MyFitnessPal	0.75	0.54	0.03	0.67
	MyDietCoach	0.77	0.80	0.57	0.86
	MyDietDiary	0.74	0.81	0.62	0.64
	MyPlate	0.86	0.76	0.56	0.82
	SparkPeople	0.89	0.85	0.64	0.94

Notes: Internal consistency (Cronbach's alpha) is excellent (≥ .90), good (.80–.89), acceptable (.70–.79), questionable (.60–.69), poor (.50–.59) and unacceptable (<.50).[38]

Inter-rater agreement indices: $r_{WG(J)}$: multi-item version of the r_{WG} index, developed by James et al. (1984).[67,68] The value represents the median of the $r_{WG(J)}$ values obtained using a uniform, normal, slight, and moderate skew null distribution, as recommended by Biemann et

al.;[69] agreement cut-offs: no agreement (<.29), weak (.03-49), moderate (.50-.69), strong (.70-.89), very strong (>.90).[65,66]

$a_{WG(J)}$*:* multi-item version of Brown and Hauenstein's a_{WG} index;[70] agreement cut-offs: not acceptable (<.59), weak (.60-69), moderate (.70-.79), strong (>.80).[70].

$A_{DMJ(adj)}$: multi-item version of the adjusted average deviation index $A_{DM(adj)}$.[71]; values above .80 represent agreement.[71]
